# Hospitalization rate in offspring of cancer survivors: a national cohort study

**DOI:** 10.1007/s11764-019-00741-5

**Published:** 2019-02-18

**Authors:** Jianguang Ji, Wuqing Huang, Jan Sundquist, Kristina Sundquist

**Affiliations:** 1 Center for Primary Health Care Research, Lund University/Region Skåne, Malmö University Hospital, CRC, floor 11, building 28, entrance 72, 205 02 Malmö, Sweden; 2 Department of Family Medicine and Community Health, Department of Population Health Science and Policy, Icahn School of Medicine at Mount Sinai, New York, NY USA

**Keywords:** Hospitalization rate, Cohort study, Epidemiology, Cancer survivor

## Abstract

**Purpose:**

The number of childbirths among cancer survivors continues to increase, but it is still largely unknown whether the children of cancer survivors might experience adverse health outcomes during the process of growing up.

**Methods:**

We identified all individuals diagnosed with cancer between 1958 and 2015 from the Swedish Cancer Registry and linked them to the Swedish Medical Birth Register to identify their offspring born between 1997 and 2015. Up to 10 children, whose parents did not have a diagnosis of cancer, were matched with the study population according to date of birth and gender.

**Results:**

By linking with the Swedish Hospital Discharge Register, we found that the hospitalization rate was 15% higher in offspring of female cancer survivors, and 16% higher in offspring of male cancer survivors as compared to matched controls. Besides an increased risk of hospitalization due to malignant neoplasms (relative risk (RR) = 1.86, 99% CI 1.70–2.04) and benign neoplasms (RR = 1.48, 99% CI 1.18–1.86), a non-significant increased risk was found for hospitalization due to infectious and parasitic disease (RR = 1.09, 99% CI 0.98–1.21), diseases of the blood and blood-forming organs and certain disorders involving the immune mechanisms (RR = 1.33, 99% CI 0.98–1.80), and diseases of the circulatory system (RR = 1.05, 99% CI 0.98–1.12).

**Conclusion:**

Our study suggests that children of cancer survivors might experience a significantly increased rate of hospitalization, which calls for further studies.

**Implications for Cancer Survivors:**

Cancer survivors might be aware that the risk of hospitalization due to various diseases might be higher in their children as compared to the normal population.

## Introduction

The number of cancer survivors continues to increase globally and this can be attributed to improved forms of treatment and enhanced rates of cancer screening [1–3]. After successfully completing treatment and when they feel healthy enough, some of these cancer survivors might plan to become parents themselves [4]. Existing evidence suggests that cancer survivors might have a lower fertility due to the side effects from cytotoxic drugs, radiation, surgery, and the disease itself, which can be temporary or permanent [5, 6]. For women, the reproductive organs, including the ovaries and the uterus, were found to be impaired by radiotherapy and chemotherapy; for men, the hypothalamic-pituitary-testicular axis might be affected [5–7].

Thus, it is plausible that parental cancer treatments might affect the health of their offspring. Available evidence suggests that cancer survivors, who are successful in becoming pregnant, might experience a range of adverse pregnancy outcomes, such as spontaneous abortion, chromosomal abnormalities, congenital malformations, preterm birth, stillbirth, and neonatal death [3, 8–13]. However, it is still largely unknown whether the children of cancer survivors might experience adverse health outcomes when they are growing up. Such knowledge is highly needed in order to guide clinical management of offspring of cancer survivors and to inform cancer survivors, who plan to become parents, of the potential health outcomes in their future offspring. This is especially pertinent in the era of continuously increased childbirths in cancer survivors [3]. In the current study, we hypothesized that the offspring of cancer survivors might experience an increased risk of clinically recognizable disease while growing up which could be measured by hospitalization rates. Until now, only one study has explored whether the risk of hospitalization in childhood might be different among offspring of cancer survivors and the general population [10]. The number of childbirths among cancer survivors quadrupled between the 1970s and 2010s in Sweden [3]. By accessing a range of Swedish national databases, we aimed to explore whether children of cancer survivors might have an increased probability of being admitted to hospital and to explore whether the association was modified by cancer sites and age at cancer diagnosis in the parents as well as which diseases that were mostly diagnosed in these children as compared to the general population.

## Material and methods

### Study population

The Ethics Committee of Lund University, Sweden, approved this study. Using the Swedish Cancer Register, we identified all patients diagnosed with cancer between 1958 and 2015; this register is maintained by the National Board of Health and Welfare [14] and covers approximately 90% of the whole country. Clinicians, pathologists, and cytologists in Sweden must report all newly diagnosed cases of cancer to the Swedish Cancer Registry. To record cancer diagnosis during the study period, we used the seventh version of the International Classification of Diseases (ICD) code.

In order to identify children of cancer survivors who were born between 1997 and 2015, we further linked to the Swedish Medical Birth Register. Since 1997, the 10th International Classification of Diseases has been used. In addition, we excluded all childbirths born within 1 year after parental cancer diagnosis in order to ensure that the child was conceived after his or her parents’ cancer diagnosis. Established in 1973, The Swedish Medical Birth Register is maintained by the National Board of Health and Welfare [15, 16] and contains information about maternal age at childbirth, height, pre-pregnancy weight, family situation (cohabiting or not cohabiting with the father-to-be), smoking habits in early pregnancy, and maternal diseases during pregnancy [17]. In this study, we excluded non-singleton pregnancies. In total, there were 6074 children identified from female cancer survivors. Up to 10 childbirths, whose mother was not diagnosed with cancer, were matched with the children of female cancer survivors according to year of birth, gender, year of birth of the parents, as well as highest education level and country of birth of the parents. Furthermore, we also identified 6441 children whose fathers were survivors of cancer and matched them with 51,512 controls.

In order to identify subsequent hospitalization after the delivery, we further linked the study population to the Swedish Hospital Discharge Register. This register was created by the National Board of Health and Welfare in 1964 and since 1987, it has included complete nationwide data and contains hospital discharge records for all individuals residing in Sweden. The Swedish Hospital Discharge Register has nearly 90% overall validity [18]. Since 1997, the primary cause of a hospitalization was listed in the discharge report and classified according to the 10th ICD codes into 24 different categories.

In addition, we further linked these children to the Cause of Death Register to identify date of death as well as the cause of death, and to the Emigration Registry to identify date of emigration. We used the individual national identification numbers to perform linkages. The ID numbers were replaced with serial numbers in order to preserve anonymity.

### Study outcomes and statistical analysis

The primary outcome was hospitalization rate, which was calculated as the total number of hospitalizations divided by the total person-years of follow-up. It should be noted that individuals might have several hospital visits during the follow-up period, and all of them contribute to cumulative hospitalization rate. We calculated person-years at risk for our study population from the date of birth to the earliest of death, emigration, or the end of the study period (December 31, 2016). Hospitalization rate was stratified by the anatomical sites of cancer in the mothers or fathers. The relative risk of hospitalization was calculated as the ratio of hospitalization rate in the study population divided by the hospitalization rate in the matched controls. To account for multiple comparisons, 99% confidence interval was used in this study. Given that cancer treatment at different ages might have different impacts on the germ cell and in vitro fertilization might play a role for the association [19], we further stratified the analyses by age at diagnosis of parental cancer and year at diagnosis. In vitro fertilization was first adopted in 1982 and it was used very rarely before 1990 in Sweden [19]. We performed all analyses using SAS version 9.2 (SAS Institute, Cary, NC, USA).

## Results

In total, there were 6074 children born after their mothers were diagnosed with cancer and 6441 children born after their fathers were diagnosed with cancer (Table 1). A total of 1322 offspring were delivered after maternal cancer diagnosis of melanoma, which accounted for 21.7% of all offspring. A total of 1777 (27.6%) offspring were delivered after paternal cancer diagnosis with testicular cancer.

**Table 1:** Characteristics of offspring of cancer survivors and their matched controls

Characteristics	Female cancer survivor				Male cancer survivor			
	Study cohorts		Matched controls		Study cohorts		Matched controls	
	*N*	Person-year	*N*	Person-year	*N*	Person-year	*N*	Person-year
Cancer site in parents								
Overall	6074	61,169	51,512	517,924	6441	64,378	54,875	546,716
Colon	336	3492	2874	29,978	240	2630	2005	21,892
Breast	312	2992	2433	23,644				
Cervical	317	3014	2638	25,067				
Ovary	215	2262	1863	19,902				
Testis					1777	17,643	840	8865
Kidney	107	1137	925	9359	153	1397	1284	11,725
Melanoma	1322	13,585	11,373	115,736	817	8319	6844	69,572
Nervous system	761	7717	6472	66,186	876	9001	7583	77,333
Thyroid gland	498	5322	4081	43,116	159	1544	1362	12,905
Endocrine glands	334	3365	2818	28,551	214	2202	1763	17,602
Bone	111	1180	924	9656	148	1600	1302	14,356
Connective tissue	161	1566	1383	13,423	183	1754	1603	15,534
Non-Hodgkin lymphoma	232	2154	1990	18,751	384	3784	3266	31,723
Hodgkin lymphoma	520	5001	4462	42,888	517	4942	4375	42,531
Leukemia	386	3566	3315	30,495	403	3812	3441	32,612
Other	462	4816	3961	41,145	463	4589	3801	38,054
< 1990	1289	15,931	9758	117,683	1458	17,641	11,425	134,893
1990–1999	2413	28,352	18,209	209,394	2588	29,937	20,656	235,589
2000+	2372	16,886	23,545	190,847	2395	16,800	22,794	176,234
Year of birth of the offspring								
< 2001	1063	18,415	8678	150,070	1091	18,958	9046	157,362
2001–2005	1659	21,248	14,348	184,088	1755	22,522	15,062	193,049
2006–2010	2091	16,535	17,879	141,921	2173	17,224	18,707	148,226
2011+	1261	4971	10,607	41,835	1422	5674	12,060	48,079

In Table 2, we present the relative risk of hospitalization in offspring of female and male cancer survivors as compared to matched controls. After 61,169 person-years of follow-up, a total of 4197 hospitalizations were found thus giving a hospitalization rate of 6.86 per 100 person-years. Compared to 51,515 matched controls, the relative risk of hospitalization was 1.15 (99% CI 1.10–1.20). For specific cancer sites in mothers, a significantly increased risk was noted when mothers were diagnosed with rectal (relative risk (RR) = 3.51), cervical (RR = 1.89), bladder (RR = 1.88), eye (RR = 3.10), nervous system (RR = 1.19), thyroid gland tumors (RR = 1.15), and with non-Hodgkin lymphoma (RR = 1.27). A total of 6441 children were delivered after their fathers were diagnosed with cancer. A total of 4477 hospitalizations were found during 64,378 person-years of follow-up thus yielding a hospitalization rate of 6.95 per 100 person-years. Compared to 54,875 matched controls, the relative risk of hospitalization was 1.16 (99% CI 1.11–1.21). For specific cancer sites in fathers, a significantly increased risk was noted when fathers were diagnosed with nose (RR = 14.42), breast (RR = 16.84), testicular (RR = 1.24), eye (RR = 4.15), kidney (RR = 1.42), and thyroid gland tumors (RR = 1.31), and with non-Hodgkin lymphoma (RR = 1.25).

**Table 2:** Relative risk of hospitalization rate in offspring of male and female cancer survivors

Site of cancer	Female cancer survivor			Male cancer survivor		
	RR	99% CI		RR	99% CI	
Overall	*1.15*	1.10	1.20	*1.16*	1.11	1.21
Upper aerodigestive tract	0.77	0.51	1.15	0.84	0.56	1.26
Salivary gland	0.57	0.31	1.03	0.70	0.27	1.81
Stomach	0.52	0.08	3.43	1.05	0.42	2.63
Small intestine	1.18	0.24	5.73	0.41	0.11	1.52
Colon	1.14	0.95	1.37	0.83	0.65	1.06
Rectum	*3.51*	1.60	7.74	0.65	0.32	1.29
Anus				0.50	0.03	8.95
Liver	1.13	0.50	2.58	0.32	0.05	2.07
Pancreas	1.81	0.45	7.36	10.00	0.76	131.97
Nose	0.83	0.06	12.22	*14.42*	9.26	22.44
Lung	0.72	0.43	1.23	1.25	0.74	2.10
Breast	1.02	0.83	1.25	*16.84*	8.46	33.51
Prostate				2.52	0.68	9.33
Testis				*1.24*	1.15	1.34
Other male genital				0.30	0.06	1.37
Cervical	*1.89*	1.63	2.20			
Endometrium	0.50	0.08	3.24			
Uterus	0.87	0.52	1.45			
Ovary	1.24	0.99	1.55			
Other female genital	1.63	0.89	2.96			
Kidney	1.07	0.78	1.48	*1.42*	1.10	1.84
Bladder	*1.88*	1.06	3.34	0.53	0.35	0.80
Melanoma	1.04	0.94	1.14	1.08	0.96	1.21
Skin	0.93	0.59	1.47	0.96	0.69	1.34
Eye	*3.10*	2.44	3.95	*4.15*	3.38	5.09
Nervous system	*1.19*	1.06	1.33	0.97	0.86	1.09
Thyroid gland	*1.15*	0.99	1.33	*1.31*	1.01	1.71
Endocrine glands	1.03	0.85	1.25	0.96	0.77	1.21
Bone	0.98	0.70	1.38	0.82	0.60	1.11
Connective tissue	0.79	0.58	1.08	1.06	0.83	1.37
Non-Hodgkin lymphoma	*1.27*	1.03	1.55	*1.25*	1.06	1.48
Hodgkin lymphoma	1.09	0.93	1.27	1.00	0.86	1.17
Myeloma				0.67	0.04	10.17
Leukemia	1.03	0.87	1.23	1.10	0.93	1.31

Italics type: 99%CI does not include 1.00

**Fig. 1:**
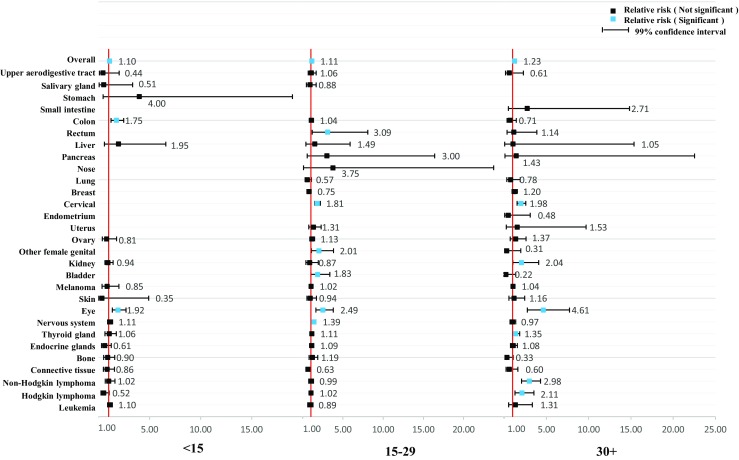
Hospitalization rate in offspring of female cancer survivors stratified by age at diagnosis

**Fig. 2:**
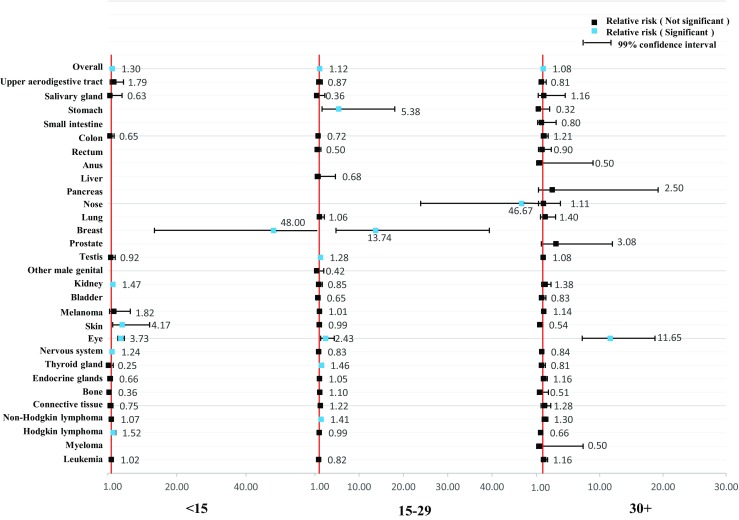
Hospitalization rate in offspring of male cancer survivors stratified by age at diagnosis

**Fig. 3:**
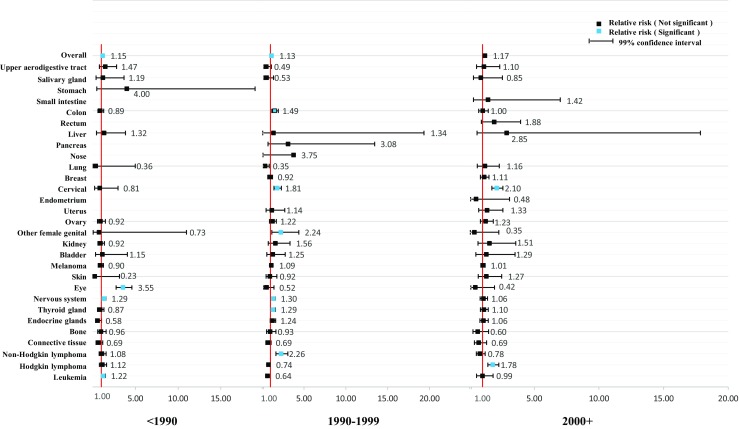
Hospitalization rate in offspring of female cancer survivors stratified by year at diagnosis of cancer

**Fig. 4:**
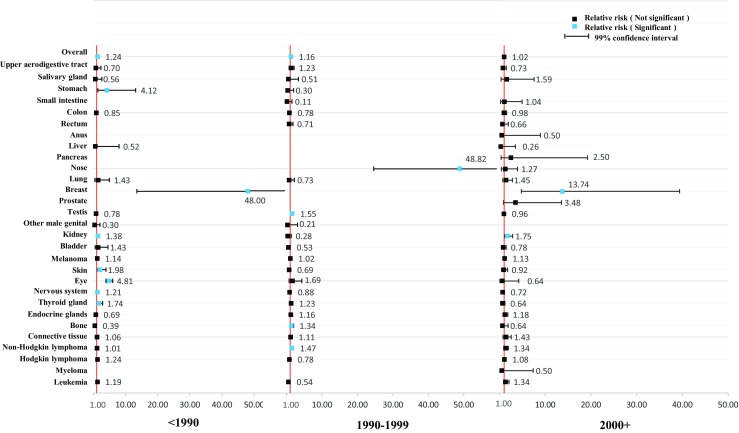
Hospitalization rate in offspring of male cancer survivors stratified by year at diagnosis of cancer

As shown in Figs. 1 and 2, we further stratified the analyses by age at diagnosis of parental cancer. The overall relative risk was relatively consistent. However, the association was stronger among female cancer survivors who were diagnosed with cancer at over 30 years of age (RR = 1.23, 99% CI = 1.13–1.35) and among male cancer survivors who were diagnosed with cancer under 15 years of age (RR = 1.30, 99% CI = 1.18–1.45). We further stratified the analyses by year at diagnosis of parental cancer in Figs. 3 and 4. The association became weaker after 1990 and was no longer significant after 2000 among the offspring of male cancer survivors, but the association was relatively consistent among the offspring of female cancer survivors.

In Table 3, we present the rate of hospitalization due to various diseases in offspring of cancer survivors. Besides an increased risk of hospitalization due to malignant neoplasms (RR = 1.86), and benign neoplasms (RR = 1.48) in the children, a marginally increased risk was found for hospitalization due to infectious and parasitic disease (RR = 1.09), diseases of the blood and blood-forming organs and certain disorders involving the immune mechanisms (RR = 1.33), and diseases of the circulatory system (RR = 1.05). A total of 19 families were diagnosed with retinoblastoma, which might be related to hereditary cancer syndromes. After exclusion of these families, the overall RR of hospitalization was 1.13 (95% CI 1.10–1.15). In addition, the RR for malignant neoplasms in the offspring was 1.37 (95% CI 1.23–1.52). We have also explored the number of bed days in hospitals for each admission with the aim to test whether surveillance bias is present. The mean bed days were 4.67 (SE = 0.12) in offspring of cancer survivors, which was significantly higher as compared to the matched controls of 4.27 (SE = 0.05); this suggests that surveillance bias might play a small role for the observed association.

**Table 3:** Hospitalization due to various diseases in offspring of cancer survivors

Disease	Study cohorts		Matched controls		RR	99% CI	
	No. of hospitalization	Rate	No. of hospitalization	Rate			
Infectious and parasitic disease	694	0.55	5398	0.51	1.09	0.98	1.21
Malignant neoplasms	545	0.44	2483	0.23	*1.86*	1.65	2.10
Benign neoplasms	88	0.07	503	0.05	*1.48*	1.10	2.00
Diseases of the blood and blood-forming organs and certain disorders involving the immune mechanism	84	0.07	535	0.05	1.33	0.98	1.80
Endocrine, nutritional and metabolic diseases	176	0.14	1595	0.15	0.94	0.76	1.15
Mental and behavioral disorders	76	0.06	648	0.06	0.99	0.73	1.36
Diseases of the nervous system	237	0.19	1988	0.19	1.01	0.85	1.21
Diseases of the eye and adnexa	152	0.12	1099	0.10	1.17	0.94	1.47
Diseases of the ear and mastoid process	57	0.05	604	0.06	0.80	0.56	1.14
Diseases of the circulatory system	1621	1.30	13,127	1.24	1.05	0.98	1.12
Diseases of the respiratory system	378	0.30	3164	0.30	1.01	0.88	1.17
Diseases of the digestive system	110	0.09	876	0.08	1.06	0.82	1.38
Diseases of the skin and subcutaneous tissue	159	0.13	1166	0.11	1.16	0.93	1.44
Diseases of the musculoskeletal system and connective tissue	285	0.23	2273	0.21	1.06	0.90	1.25
Congenital malformations, deformations and chromosomal abnormalities	616	0.49	4947	0.47	1.06	0.95	1.18
Symptoms, signs and abnormal clinical and laboratory findings, not elsewhere classified	617	0.49	4913	0.46	1.06	0.95	1.19
Injury, poisoning and certain other consequences of external causes	179	0.14	1355	0.13	1.12	0.91	1.38
Others	2501	2.00	16,674	1.57	*1.27*	1.20	1.34

## Discussion

In this population-based study, which is to our knowledge the largest study on this topic, we found that children of cancer survivors experienced a significantly higher hospitalization rate due to various diseases. We also found that, irrespective of maternal or paternal cancer survivors, the increased risk of hospitalization was largely consistent. However, the relative risk of hospitalization was stronger when mothers were diagnosed with adult cancer and when fathers were diagnosed with childhood cancer.

When interpreting the current findings, it is important to factor in a few strengths and limitations. The present study has a number of strengths, which include its nationwide coverage and the diagnosis of patients by specialists in a country of high medical standards. The population-based approach allowed a complete identification of cancer survivors, as well as their children born after their diagnosis of cancer. Our study is free of recall bias as we used registry-based data, as opposed to self-reported data. However, it should be noted that only around 12,000 children were identified from parental cancer survivors even from this nationwide study that uses data with complete coverage of the whole of Sweden. We thus have limited study power to stratify the observed findings by anatomic site or other characteristics of the parental cancer. It is highly necessary to implement international collaborations going forward to increase the power of identifying the true association and to exclude chance findings. One limitation of this study is that some risk factors, which might be associated with hospitalization due to various diseases, were not available in our databases; this may partly confound our observations. However, we matched the study population with controls by date of birth and gender, and limited the study population for children born after 1997. The maximum age of the study population was less than 20 years old at the end of the study; thus, some common behavioral risk factors such as cigarette smoking and alcohol drinking might be less prevalent in the study population [20, 21]. A lack of accurate assessment of treatment information was another limitation. It should be noted that children of cancer survivors might experience an increased hospitalization rate due to surveillance bias, i.e., parents that are cancer survivors might be more concerned about their children’s health status than other parents. However, we found that the number of bed days in hospitals among offspring of cancer survivors was significantly higher as compared to the matched controls, which suggests that surveillance bias might play a small role for the observed association.

It is well-known that patients with cancer who received various treatments have a risk of developing various long-term side effects, and the long-term side effects might depend on the treatments that patients have received as well as the area of the body exposed to the treatments [22–24] Available evidence suggests that an increased incidence of adverse pregnancy outcomes was noted, such as preterm birth and low birth weight, which was strongly related to long-term growth damage and morbidity [3, 13, 25–29]. Our study provides evidence that children of cancer survivors have an increased rate of hospitalization as compared to the general population.

Existing evidence suggests that, exposure to cancer treatment might affect the health of the offspring not only through DNA mutation but also through epigenetic modifications, due to the direct effect from damage of the ovaries or testis, or indirect effects from injury of the hypothalamic-pituitary-gonadal axis [11, 12, 30–32]. Growing evidence from recent experimental animal-based studies indicates that epi-mutation encoded in sperm or oocytes are heritable and may affect offspring phenotypes [33, 34]. The germ cells can develop various epi-mutations when exposed to environmental toxicants or stress during spermatogenesis or ovogenesis; this might affect gene expression although it does not affect the actual base pair sequence of DNA. As a common epigenetic mechanism, DNA methylation regions in the sperm of patients with childhood osteosarcoma, which were exposed to chemotherapy, showed a significant difference as compared to male patients without the same exposure [35]. Furthermore, even 10 years after the exposure, the difference remained significant. Based on these findings, it is reasonable to infer that chemotherapy exposure can promote epigenetic alterations that persist later in life, and such exposure has the potential to promote epigenetic inheritance to the next generation.

Although the increased overall risk of hospitalization was largely consistent among the offspring of female or male cancer survivors, a stronger association was found among the offspring of maternal adult cancer survivors and paternal childhood cancer survivors. Female childhood cancer patients might suffer from permanent ovarian failure, leading to non-fertility [7]. For those who are able to give birth, the ovarian reserve among younger females is more robust than that of older women due to greater complement of primordial follicles; it is thus reasonable that younger female cancer survivors are more resistant to damage to the ovaries from chemicals and radiation [36–39]. Cancer treatments to male patients might affect the hypothalamic-pituitary-testicular axis. Radiation of testicular, crania or total body and orchiectomy, and gonadotoxic chemicals tend to affect spermatogenesis, sperm function and cause hypoandrogenism [39–41]. Pubertal status was suggested to be associated with gonadotoxicity caused by radiation, which means that the reproductive system in males before puberty might be more sensitive to radiation than when treated after puberty [39, 41].

It is known that common cancers have familial aggregation [42–45], which might explain that children of cancer survivors have an increased rate of hospitalization due to cancer. Besides cancer, a marginally increased rate of hospitalization due to infectious diseases or diseases due to immune deficiency was noted in children of cancer survivors; this suggests that children of cancer survivors might be associated with an abnormal immune function. A previous study found that cancer survivors had a high incidence of various autoimmune diseases [46], suggesting immune functions in these cancer survivors has changed because of various treatments. One possible reason that might explain the inconsistent results between the present and the Danish study is that our study aimed to look at the cumulative hospitalization rate, which meant that one individual might have more than one hospitalization during the follow-up period. However, the Danish study examined only the incidence of hospitalization, and the follow-up will be stopped after the first hospitalization. By removing possible hereditary cancer syndromes, the relative risk of cancer (RR = 1.38) was largely consistent with the published data from the Nordic Cancer Registries (SIR = 1.3), suggesting that the study outcomes were relatively reliable in this population-based cohort study.

## Conclusion

In summary, the present study shows that the hospitalization rates in children of cancer survivors are significantly higher as compared to the general population. These findings supported our hypothesis that offspring of cancer survivors might experience an increased risk of clinically recognizable disease when they grow up. However, the underlying mechanisms need to be explored further.
